# Overcoming challenges in real‐world evidence generation: An example from an Adult Medical Care Coordination program

**DOI:** 10.1002/lrh2.10430

**Published:** 2024-05-22

**Authors:** Samuel T. Savitz, Michelle A. Lampman, Shealeigh A. Inselman, Ruchita Dholakia, Vicki L. Hunt, Angela B. Mattson, Robert J. Stroebel, Pamela J. McCabe, Stephanie G. Witwer, Bijan J. Borah

**Affiliations:** ^1^ Robert D. and Patricia E. Kern Center for the Science of Health Care Delivery, Mayo Clinic Rochester Minnesota USA; ^2^ Division of Health Care Delivery Research Mayo Clinic Rochester Minnesota USA; ^3^ Division of Community Internal Medicine, Geriatrics, and Palliative Care Mayo Clinic Rochester Minnesota USA; ^4^ Department of Nursing Mayo Clinic Rochester Minnesota USA

**Keywords:** care transitions, case management, patient readmission, pragmatic clinical trial

## Abstract

The Adult Medical Care Coordination program (“the program”) was implemented at Mayo Clinic to promote patient self‐management and improve 30‐day unplanned readmission for patients with high risk for readmission after hospital discharge. This study aimed to evaluate the impact of the program compared to usual care using a pragmatic, stepped wedge cluster randomized trial (“stepped wedge trial”). However, several challenges arose including large differences between the study arms. Our goal is to describe the challenges and present lessons learned on how to overcome such challenges and generate evidence to support practice decisions. We describe the challenges encountered during the trial, the approach to addressing these challenges, and lessons learned for other learning health system researchers facing similar challenges. The trial experienced several challenges in implementation including several clinics dropping from the study and care disruptions due to COVID‐19. Additionally, there were large differences in the patient population between the program and usual care arms. For example, the mean age was 76.8 for the program and 68.1 for usual care. Due to these differences, we adapted the methods using the propensity score matching approach that is traditionally applied to observational designs and adjusted for differences in observable characteristics. When conducting pragmatic research, researchers will encounter factors beyond their control that may introduce bias. The lessons learned include the need to weigh the tradeoffs of pragmatic design elements and the potential value of adaptive designs for pragmatic trials. Applying these lessons would promote the successful generation of evidence that informs practice decisions.

## 1 BACKGROUND

A key activity of learning health systems is to evaluate interventions to inform care delivery. However, challenges often arise due to external factors affecting care delivery, difficulties in implementation, and the need to identify an appropriate comparison group. Overcoming these challenges to produce evidence that informs the continuation and modification of health system interventions is critical.

In this commentary, we describe our experience with an evaluation of the Adult Medical Care Coordination program (“the program”) at Mayo Clinic. The program was implemented to promote patient self‐management and reduce unnecessary hospital readmissions. The program is an adaptation of the Coleman Care Transitions Intervention.^1^ RN care coordinators provided coaching to patients with one home visit and regular phone calls up to 6 months post‐discharge. Eligible patients had to: be paneled in Mayo Clinic primary care; be aged ≥18; be discharged from the hospital to home or assisted living; have ≥2 chronic conditions; and have a LACE+^2^ readmission risk score of ≥59 (see Table S1 for full criteria). The primary outcome was 30‐day unplanned readmissions. The goal was to evaluate the program to inform the evolution of care models to reduce unnecessary readmissions and support care transitions.

## 2 PLANNED STUDY DESIGN

The analysis was designed as a pragmatic,^3^ stepped wedge cluster randomized trial (“stepped wedge trial”).^4^ Stepped wedge trials are a type of trial in which clusters (e.g., clinics) are randomized to receive an intervention in a series of planned steps or tranches. Our original trial design applied the stepped wedge trial to three study arms: the program, remote patient monitoring (“remote monitoring”), and usual care. The trial was designed to compare the program and remote monitoring with usual care, but not the program and remote monitoring with each other. The clusters included Mayo Clinic primary care clinics in Minnesota and Wisconsin that would implement the program or remote monitoring in three planned steps. The baseline phase before the trial was identified retrospectively without recruitment into the study. After the initiation of the trial, patients in usual care had to provide informed consent. The trial was scheduled to begin in January 2020 and proceed for 9 months. The implementation of the program employed several implementation strategies such as providing training for the RN care coordinators.

We selected a pragmatic, stepped wedge trial design for several reasons. First, pragmatic trials minimize disruption to the health system and provide a more realistic estimate of the impact of an intervention on real‐world practice than traditional randomized controlled trials (RCTs).^3^ Second, the stepped wedge trial design allows for all clusters to contribute observations to both control and treatment arms. Additionally, it ensures that all clinics will eventually receive the program. Third, rolling out the intervention by cluster accounts for possible spillover effects that may occur across patients in the same cluster.^4^ Although there are many advantages of pragmatic trials and stepped wedge trials, we recognized that there are also challenges with these designs. For pragmatic trials, design choices that make the trial more pragmatic may increase the risk of bias compared to the tightly controlled conditions of a traditional RCT.^3^ Common challenges of stepped wedge trial include confounding with time since there are more intervention units during later periods of the trial, selection bias if individual recruitment is required and blinding is infeasible,^4^ and logistical challenges that lead to implementation problems.^5^ On balance, we felt that the results of a pragmatic, stepped wedge trial would be most informative to the practice.

## 3 CHALLENGES EXPERIENCED

**FIGURE 1:**
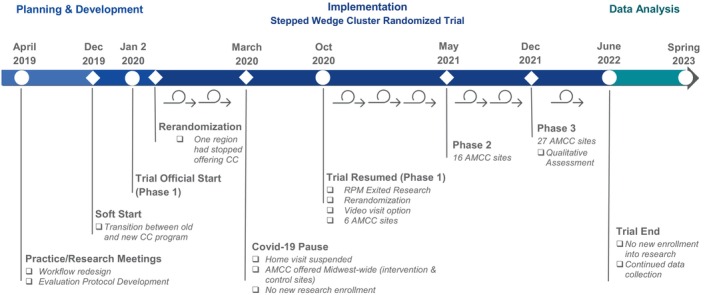
Trial timeline. AMCC, adult medical care coordination; CC, care coordination; RPM, remote patient monitoring.

We experienced challenges that affected the rollout of the intervention (Figure 1). First, one of the regions stopped offering care coordination after randomization and the clinics were rerandomized after the start of the trial. All of the clinics in this region were assigned to eventually receive remote monitoring. However, since remote monitoring and the program were not being directly compared, this would not bias the results. Second, the trial began in January 2020 and the COVID‐19 pandemic began affecting care delivery in early March. We paused the trial due to the onset of the pandemic and suspended home visits for the program. During the pause, the program was offered at all clinics except for the region that discontinued care coordination, and remote monitoring services were redirected to support patients with COVID‐19. Upon resumption in October 2020, we discontinued the remote monitoring arm due to the continued need to focus on COVID‐19, and rerandomized the clinics in the second and third tranches. The region that discontinued care coordination was dropped from the trial and the program was ultimately implemented at 27 clinics. When the trial resumed, there was an option to do the initial program visit by video rather than exclusively at home given concerns about COVID‐19 exposure.

There were also challenges in recruitment that led to differences (Table S2). Patients in the program arm were older (mean age: 76.8 program arm; 68.1 usual care) and had a higher LACE+ readmission risk score^2^ (74.0 program arm; 70.5 usual care). Patients in the program are also had more Elixhauser comorbidities and were more likely to have each chronic condition.

These differences were likely due to variations in how patients were recruited and the consent requirements. Patients recruited to the program would be asked to participate but did not have to provide research consent for this study since the program was a standard care offering in these clinics. However, patients receiving care in Minnesota would have to provide general research authorization under Minnesota law, which is required for any research study using medical records from patients in the state.^6^ In contrast, patients recruited to usual care were not offered a program but had to provide research consent for this particular study. As such, consent bias^7^ may have differentially affected the two arms. Consent bias has been shown to lead to differences in demographics and health status in other trials.^8^ Additionally, the potential to benefit from the program may have affected the decision to participate in the program.

There also appeared to be more conservative screening practices for usual care. This difference arose in part because the clinically trained RN care coordinators selecting patients for the program were different from the non‐clinically trained study coordinators selecting patients for usual care. We made this decision to minimize the disruption from the pragmatic trial on the routine operations of clinical practice. For example, there was an exclusion criterion for patients with dementia or moderate to severe cognitive impairment. This criterion was applied to usual care to ensure that patients could provide informed consent and respond to a patient‐reported outcome measure. However, for the program, the criterion was less restrictive, which allowed patients with mild dementia or cognitive impairment to be included if a caregiver was able to work with the RN care coordinator. This difference helps explain the higher prevalence of dementia in the program (6.3%) compared to usual care (1.3%). More generally, the study coordinators seemed to apply the inclusion and exclusion criteria more conservatively. There was also turnover among the study coordinators during the trial. We were aware that there would be some differences due to the program being less restrictive and planned to exclude patients such as those with dementia or cognitive impairment. However, we found that excluding these patients was insufficient to address differences.

Despite these challenges, it was important to determine the impact of the program to inform the operational decisions about the program. To address the observed differences, we analyzed the data using propensity score matching (see Technical Appendix).^9^ After identifying the matches, we then proceeded to evaluate the differences in 30‐day unplanned readmissions and secondary outcomes (e.g., 90‐day unplanned readmissions) using the matched sample and the findings will appear in future work. These results have been presented to practice leadership and are being used to make operational decisions about the program.

## 4 CHALLENGES INHERENT TO PRAGMATIC RESEARCH

The challenges we experienced illustrate the tradeoffs involved in conducting pragmatic research in a learning health system. In practice, almost no trials are fully pragmatic in all domains as measured by the PRECIS‐2 (PRagmatic Explanatory Continuum Indicator Summary) tool.^10^ Choosing to be more or less pragmatic reflects tradeoffs with respect to the relevancy of findings to decision‐makers, the burden on the health system and patients, and potential sources of bias. While it is a common misconception that pragmatic elements of trial design necessarily sacrifice internal validity,^11^ it is often the case that making a trial more pragmatic will increase the risk of some forms of bias. In our case, we had different staff who made determinations on inclusion in the two study arms. In addition, the inclusion criteria were applied more strictly in the usual care arm compared to the program arm. If we had the same staff determining inclusion for both arms and made the criteria as similar as possible, then we likely would not have experienced the observed differences and would not have needed to adapt the methodological approach. However, efforts to make recruitment more consistent would have made the trial less pragmatic with respect to aligning recruitment with clinical practice operating as usual. As such, it is important to consider whether the benefits of keeping the trial more pragmatic outweigh the risks of differences due to issues like differential recruitment. It may also be possible to keep the trial pragmatic in this area and improve consistency by evaluating how the criteria are being applied at regular intervals so that feedback can be provided to staff to ensure they apply the criteria consistently across the study arms.

## 5 ADAPTIVE TRIALS AS A STRATEGY TO PROTECT AGAINST THESE CHALLENGES

The process of overcoming the challenges illustrates the value of considering pre‐specified adaptive designs when conducting pragmatic research. The main objective of our work was to generate the best evidence possible to inform the decision for the continuation of the program. This goal led to unplanned adaptations to the original trial design to account for unanticipated changes across the clinical setting. We also adapted the analytical approach to account for the large differences across the two arms. While adaptations that deviate from the protocol should not be made without good cause, we needed to make the adaptations to complete the trial (such as rerandomization after a region stopped providing care coordination), estimate the impact of the program, and be responsive to the needs of the practice leadership.

Given that unplanned adaptations can undermine the validity of a trial,^12^ it would be prudent to use an adaptive study design to incorporate pre‐planned strategies for identifying and mitigating the common challenges for pragmatic stepped wedge trials. Adaptive designs use pre‐specified rules for protocol‐consistent adaptations, including eliminating study arms and focusing recruitment on patients more likely to benefit.^13^ While the adaptive trial design has historically been more focused on increasing study efficiency for traditional RCTs,^13^ they also have the potential to be applied to pragmatic stepped wedge trials to protect against threats to validity. For example, we could have pre‐specified conducting an interim analysis to ascertain if there were differences between the two arms at an early stage and take corrective actions to study recruitment earlier. To make this decision more objective, it would be necessary to pre‐specify criteria, including the variables of interest, the metrics for detecting differences, the threshold for those metrics, and the specific corrective actions to be taken. By pre‐specifying adaptations and rules for enacting the adaptations, researchers can make adaptations without deviating from the protocol and minimize the risk of introducing bias.

## 6 CONCLUSIONS

This experience illustrates the challenges that can arise with pragmatic stepped wedge trials. Many of the challenges reflected factors that were beyond our control including the COVID‐19 pandemic and one of the sites no longer offering care coordination. While we had more control over recruitment, we still faced challenges that stemmed from the aim to make the trial less disruptive to the health system, and, hence, more pragmatic. We recommend researchers consider the tradeoffs and choices with respect to allowing flexibility for clinical practice to operate as usual and the need to protect against common forms of bias. Since there are potential challenges inherent to the design of pragmatic stepped wedge trials, it is prudent to take preemptive steps to protect against these threats, such as pre‐specifying adaptations for actions that will be taken if particular challenges occur. Doing so will aid in generating critical evidence to inform practice decisions in the face of commonly occurring challenges.

## CONFLICT OF INTEREST STATEMENT

Dr. Borah has received consulting fees from Exact Sciences and Boehringer Ingelheim for work not related to this manuscript. The other authors have no conflicts to report.

## Supporting information


**Table S1.** Detailed inclusion and exclusion criteria for adult medical care coordination.

**Table S2.** Descriptive statistics.

Technical Appendix.
