# Supplementary material for: Overcoming challenges in real‐world evidence generation: An example from an Adult Medical Care Coordination program
Source: Learn Health Syst. 2024 May 22;8(Suppl 1):e10430. doi: 10.1002/lrh2.10430 (PMC11488116; doi:10.1002/lrh2.10430)
Supplement: Supplementary file 1 — Table S1. Detailed inclusion and exclusion criteria for adult medical care coordination. [file LRH2-8-e10430-s003.docx]

Supplementary Table 1: Detailed Inclusion and Exclusion Criteria for Adult Medical Care Coordination

| Inclusion Criteria | Age ≥18 years |
| --- | --- |
|  | Discharged from a Mayo Clinic or non-Mayo Clinic hospital (inpatient stay) or hospital observation status in the past 7 days. |
|  | LACE+ score ≥ 59 and at least two chronic conditions. |
|  | English speaking. |
|  | Normal cognitive function. Mild dementia or mild cognitive impairment is allowed if a caregiver is able to work with the care coordinator and patient during program enrollment. |
|  | Mayo Clinic or Mayo Clinic Health System provider managing the patient’s care (e.g. primary care). Patient is paneled to a Mayo Clinic MD/NP/PA. |
|  | Access to and ability to communicate via telephone and/or video (either patient or caregiver). |
|  | Discharge to home (residential dwelling) or assisted living setting and available by telephone and/or video. |
|  | Patient and/or caregiver are interested and agree to participate in the care coordination program. |
|  | If a patient is eligible for Integrated Behavioral Health care coordination or enrolled in Integrated Behavioral Health care coordination – Integrated Behavioral Health and medical care coordinators should discuss further with supervising Integrated Behavioral Health psychiatrist for which program takes precedence for the patient; dual enrollment not a hard exclusion. |
| Exclusion Criteria | Index hospitalization, but discharged to rehabilitation/skilled nursing facility/transitional care unit, assisted living memory unit or group home before going home. |
|  | ED Observation Status. |
|  | Life expectancy < 6 months or enrolled in hospice or palliative care programs. |
|  | Permanently living in a long-term care facility or a memory unit of an assisted living facility. |
|  | Chronic pain syndrome if chronic pain is the only diagnosis and no chronic medical condition exists. |
|  | Patients with a serious and persistent mental health disorder or severe treatment interfering behavior that require a higher level of service than is available at the patient’s clinic. |
|  | Pre transplant patients with evidence of actively being followed by transplant &/or transplant Care Coordinator is assigned. Excluded until one year post transplant. |
|  | Patients actively receiving dialysis. |
|  | Hematology/oncology patients with evidence of active management from specialty. |
|  | Patient is unwilling to sign a Release of Information – allows those providing care, internal and external to be actively involved in patient’s care coordination). |
|  | Active substance abuse (excluding tobacco) for which the patient is unwilling to address. |
|  | Patients with active Tuberculosis or active COVID diagnosis at discharge. |
|  | Patient is pregnant. |
|  | Patient has a Violent Patient Flag noted in Epic. |
|  | Patient declines home or video visit by care coordinator. |
|  | Psychiatric hospital admission. |
|  | Dementia or moderate to severe cognitive impairment. |
|  | Patient is already enrolled in Remote Patient Monitoring, Care Transitions Program, Palliative Care Homebound Program, or Primary Care House Calls Program. |
|  | Religious Sisterhood living at Mayo Clinic Hospital - St. Mary’s Campus or Assisi Heights (Rochester only). |

Note: The criteria in this table were used in the course of clinical practice to identify eligible patients for the program. While we attempted to replicate the criteria for the comparison group, some criteria were not available from the EHR.
