# Supplementary material for: Overcoming challenges in real‐world evidence generation: An example from an Adult Medical Care Coordination program
Source: Learn Health Syst. 2024 May 22;8(Suppl 1):e10430. doi: 10.1002/lrh2.10430 (PMC11488116; doi:10.1002/lrh2.10430)
Supplement: Supplementary file 2 — Table S2. Descriptive statistics. [file LRH2-8-e10430-s001.docx]

Supplementary Table 2: Descriptive Statistics

| **Patient Characteristics** | | | | | | | | | |
| --- | --- | --- | --- | --- | --- | --- | --- | --- | --- |
|  | | **Treatment Arm** **(N=923)** | | **Usual Care** **(N=1024)**​ | | **Total** **(N=1947)** | | **P-value** | |
| **Age** | |  | |  | |  | | <.0001^2^ | |
| Mean (SD) | | 76.8 (11.72)​ | | 68.1 (14.02)​ | | 72.2 (13.69)​ | | ​ | |
| Median (IQR) | | 78.0 (70.0, 85.0)​ | | 70.0 (61.0, 78.0)​ | | 74.0 (65.0, 82.0)​ | | ​ | |
| **Gender, n (%)** | | ​ | | ​ | | ​ | | 0.0056^1^​ | |
| Female**​** | | 479 (51.9%)​ | | 467 (45.6%)​ | | 946 (48.6%)​ | | ​ | |
| Male**​** | | 444 (48.1%)​ | | 557 (54.4%)​ | | 1001 (51.4%)​ | | ​ | |
| **Race, n (%)​** | | ​ | | ​ | | ​ | | 0.2922^1^​ | |
| Black or African American**​** | | 14 (1.5%)​ | | 13 (1.3%)​ | | 27 (1.4%)​ | | ​ | |
| Asian or Pacific Islander**​** | | 10 (1.1%)​ | | 4 (0.4%)​ | | 14 (0.7%)​ | | ​ | |
| White**​** | | 888 (96.3%)​ | | 997 (97.5%)​ | | 1885 (96.9%)​ | | ​ | |
| Other or Choose Not to Disclose**​** | | 10 (1.1%)​ | | 9 (0.9%)​ | | 19 (1.0%)​ | | ​ | |
| Missing**​** | | 1​ | | 1​ | | 2​ | | ​ | |
| **Ethnicity, n (%)​** | | ​ | | ​ | | ​ | | 0.7822^1^​ | |
| Not Hispanic or Latino**​** | | 895 (97.0%)​ | | 998 (97.5%)​ | | 1893 (97.2%)​ | | ​ | |
| Hispanic or Latino**​** | | 17 (1.8%)​ | | 15 (1.5%)​ | | 32 (1.6%)​ | | ​ | |
| Other or Choose Not to Disclose**​** | | 11 (1.2%)​ | | 11 (1.1%)​ | | 22 (1.1%)​ | | ​ | |
| Rurality (2 levels), n (%)**​** | | ​ | | ​ | | ​ | | <.0001^1^ | |
| Urban**​** | | 627 (67.9%)​ | | 535 (52.2%)​ | | 1162 (59.7%)​ | | ​ | |
| Rural**​** | | 296 (32.1%)​ | | 489 (47.8%)​ | | 785 (40.3%)​ | | ​ | |
| LACE+ Score**​** | | ​ | | ​ | | ​ | | <.0001^2^​ | |
| Mean (SD)**​** | | 74.0 (7.50)​ | | 70.5 (7.83)​ | | 72.0 (7.88)​ | | ​ | |
| Median (IQR)**​** | | 76.0 (67.0, 80.0)​ | | 70.0 (63.0, 77.0)​ | | 74.0 (64.0, 79.0)​ | | ​ | |
| Number of **Elixhauser** **Comorbidities**, n (%)**​** | | ​ | | ​ | | ​ | | <.0001^1^​ | |
| 1-5**​** | | 271 (29.4%) | | 512 (50.8%) | | 783 (40.6%) | |  | |
| 6-10**​** | | 448 (48.5%) | | 406 (40.3%) | | 854 (44.2%) | |  | |
| 11-15 | | 179 (19.4%) | | 87 (8.6%) | | 266 (13.8%) | |  | |
| 16+ | | 25 (2.7%) | | 2 (0.2%) | | 27 (1.4%) | |  | |
| Proportion of Patients with Key Conditions, n (%) | |  | |  | |  | |  | |
| Coronary Artery Disease | | 548 (59.4%)​ | | 441 (43.1%)​ | | 989 (50.8%)​ | | <.0001^1^​ | |
| Congestive Heart Failure | | 503 (54.5%)​ | | 359 (35.1%)​ | | 862 (44.3%)​ | | <.0001^1^​ | |
| Dementia | | 58 (6.3%) | | 13 (1.3%) | | 71 (3.7%) | | <.0001^1^ | |
| Atrial Fibrillation | | 462 (50.1%)​ | | 385 (37.6%)​ | | 847 (43.5%)​ | | <.0001^1^​ | |
| Diabetes Mellitus | | 414 (44.9%)​ | | 352 (34.4%)​ | | 766 (39.3%)​ | | <.0001^1^​ | |
| Chronic Kidney Disease | | 397 (43.0%)​ | | 255 (24.9%)​ | | 652 (33.5%)​ | | <.0001^1^​ | |
| Chronic Obstructive Pulmonary Disease | | 285 (30.9%)​ | | 231 (22.6%)​ | | 516 (26.5%)​ | | <.0001^1^​ | |
| Active Malignancy | | 158 (17.1%)​ | | 123 (12.0%)​ | | 281 (14.4%)​ | | 0.0014^1^​ | |
| End-Stage Renal Disease (dialysis)**​** | | 18 (2.0%)​ | | 8 (0.8%)​ | | 26 (1.3%)​ | | 0.0249^1^​ | |
| Morbid Obesity**​** | | 16 (1.7%)​ | | 9 (0.9%)​ | | 25 (1.3%)​ | | 0.0944^1^​ | |
| ^1^Chi-Square p-value; ^2^Kruskal-Wallis p-value;**​** | | | | | | | | | |

SD: standard deviation; IQR: interquartile range
