# Supplementary material for: Overcoming challenges in real‐world evidence generation: An example from an Adult Medical Care Coordination program
Source: Learn Health Syst. 2024 May 22;8(Suppl 1):e10430. doi: 10.1002/lrh2.10430 (PMC11488116; doi:10.1002/lrh2.10430)
Supplement: Supplementary file 3 — Technical Appendix. [file LRH2-8-e10430-s002.docx]

# TECHNICAL APPENDIX

We used 1:1 propensity score matching to address the differences we observed. Propensity score matching balances individual characteristics across levels of treatment. First, a propensity score is calculated using an approach such as logistic regression with the outcome being receipt of treatment. The model includes the variables to be balanced between treatment and control arms. Then, the propensity score is used to create matches by identifying individuals in each arm who have similar propensity score values. To ensure that we had enough matches and to address the bias in the usual care arm, we used a retrospective cohort of individuals not in the trial as potential matches for the program arm. Otherwise, we may not have identified enough matches given the underlying differences between the two arms. The main advantages of using propensity score matching is that it can reduce the differences on observed characteristics while at the same time it is more straightforward to explain the design than other approaches like propensity score weighting. The main limitation is that propensity score matching only adjusts for observable characteristics and there may still be differences for unmeasured characteristics. After identifying the matches, we found that the differences on observed characteristics were addressed for most variables. The residual differences were adjusted through regression modeling of the outcomes of interest.
